# Epigenetic Modulators: Role of Gut Microbiome in Transformation of Nutrient Bioactives and Host Gene Regulation

**DOI:** 10.3390/cells15110957

**Published:** 2026-05-22

**Authors:** Hadeel Edkaidek, Divakar Dahiya, Poonam Singh Nigam

**Affiliations:** 1 Department of Food Processing, Faculty of Agriculture, Palestine Technical University-Al-Aroub Branch, Hebron P625, Palestine; 2 Basingstoke and North Hampshire Hospital, Basingstoke RG24 9NA, UK; 3 Biomedical Sciences Research Institute, Ulster University, Coleraine BT52 1SA, UK

**Keywords:** nutrients, gut microbiome, epigenetics, polyphenols, short-chain fatty acids, DNA methylation, histone acetylation, precision nutrition

## Abstract

Biological activity of diets consisting of dietary fibers, peptides and polyphenols is largely mediated by the gut microbiota, which converts these compounds into bioactive metabolites. This review examines the microbiota–epigenome axis, highlighting gut microbiota-derived metabolites, including short-chain fatty acids (SCFAs), urolithins, and phenolic acids, that modulate host gene expression through DNA methylation, histone modifications, and non-coding RNA regulation. Current evidence from molecular and microbiome studies indicates that these metabolites influence key metabolic and inflammatory pathways, including lipid absorption via CD36, SIRT1 activation, and one-carbon metabolism involving folate and S-adenosylmethionine (SAM). Inter-individual variability in metabolic responses is associated with differences in microbial composition and metabotypes, which determine the magnitude of epigenetic regulation. Furthermore, dietary polyphenols derived from pomegranate, berries, tea, cocoa, and grapes are shown to modulate gut microbiota composition and enhance epigenetic effects. A “butyrate–polyphenol synergy” model is proposed, in which combined microbial metabolites optimize host epigenetic programming. Overall, agri-food by-products are suggested to function as modulators of the host epigenetic landscape, providing a framework for microbiome-targeted dietary strategies to improve metabolic and inflammatory health.

## 1. Introduction

A balanced diet is a potential source of bioactive compounds capable of improving human nutrition and sustaining general well-being [1]. The efficiency of bioactive molecules, including agriculture-sourced prebiotics and nutraceuticals containing probiotics, has been well studied [2]. Furthermore, it is important to study the epigenetic mechanisms through which their microbially transformed metabolites modulate host gene expression. Specifically, the intricate ‘cross-talk’ between gut microbiota-derived metabolites and host chromatin remodeling remains to be fully understood, limiting our ability to harness nutraceuticals as targeted precision nutrition tools [3]. Addressing these challenges requires an integrated approach that couples compositional characterization with functional and bioactive assessments [4].

Building on the potential of our diet as a source of bioactive compounds for health, it is increasingly recognized that their biological effects are mediated through interactions with the gut microbiota. The microbial communities residing in the gastrointestinal tract coevolve with the host and play a central role in transforming complex dietary components into metabolites that influence host physiology [5]. While host genetics, age, diet, and lifestyle shape microbial composition [6,7]. Emerging evidence indicates that hosts can actively modulate their microbiota through epigenetic mechanisms, adjusting gene expression without altering the underlying DNA sequence [8]. This dynamic crosstalk between the host epigenome and gut microbiota not only supports intestinal homeostasis but also allows adaptive responses to environmental or dietary changes. Nevertheless, despite growing insights into host–microbiota interactions, the specific contribution of diet-derived bioactive compounds to microbiota-mediated epigenetic regulation remains largely unexplored, representing a critical gap in current research [9].

The ultimate influence of diet-derived bioactive compounds on host health is largely mediated through microbiota-derived metabolites (MDMs), which act as potent modulators of the host epigenome [10]. These metabolites, including short-chain fatty acids, amino acid derivatives, and microbial vitamins, can influence DNA methylation, histone modification, and non-coding RNA regulation, thereby shaping gene expression across multiple tissues [10]. Through the recently proposed microbiota-derived metabolites–epigenetic (MDME) axis, MDMs serve as intermediates connecting microbial metabolism to systemic host physiology, establishing communication along the gut-liver and gut–brain axes [9]. Experimental models highlight the bidirectional nature of this regulation: modulation of epigenetic enzymes such as SIRT1, HDAC6, and KDM5 alters microbiota composition and metabolite production, while microbial metabolites, in turn, impact epigenetic landscapes and host phenotypes [11]. Despite these insights, the precise contribution of diet-derived MDMs, particularly those generated from nutrition, to epigenetic programming remains underexplored, representing a critical gap for future research [9,11].

While several reviews have explored the general link between the gut microbiota and epigenetic mechanisms, this work is unique in its focus on the synergistic transformation of diverse nutrient classes—specifically dietary fibers, bioactive peptides, and polyphenols—into a collective pool of epigenetic modulators. Unlike previous literature that often treats these components in isolation, we provide a holistic integration of how the microbiota–epigenome axis serves as a metabolic sensor for precision nutrition. Furthermore, this review uniquely discusses the clinical relevance of microbial metabolites like urolithins as systemic signaling molecules that bridge the gap between dietary intake and long-term host gene regulation through “epigenetic memory” in metabolic and inflammatory diseases.

The primary objective of this review is to interpret the role of diet-derived bioactive compounds in modulating the host epigenome through interactions with the gut microbiota. Specifically, we aim to

(i) Examine the metabolic transformation of these bioactives by gut microbiota;
(ii) Highlight the mechanisms by which MDMs influence epigenetic modifications, including DNA methylation, histone modification, and non-coding RNA regulation;
(iii) Discuss current gaps in understanding the contribution of MDMs from agri-food by-products to host gene regulation and systemic health. By integrating molecular, microbial, and nutritional perspectives, this review seeks to provide a comprehensive framework for harnessing agri-food by-products in the development of functional foods, nutraceuticals, and microbiota-targeted strategies for health promotion.

## 2. Bioavailability Challenges and Role of Gut Microbiota

Dietary bioactive compounds exhibit limited bioavailability, with only a minor fraction absorbed in their native form, reflecting not only physicochemical constraints but also the dependence on microbial metabolism for functional activation [12,13]. In this context, the gut microbiota operates as a metabolic interface that is involved in the conversion of structurally complex substrates into low-molecular-weight bioactive metabolites through enzymatic transformations such as hydrolysis and reduction [14,15].

However, this conversion is highly variable and strongly dependent on microbial composition and enzymatic capacity, introducing significant interindividual differences in metabolic outputs and associations with biological responses [6]. This challenges the conventional host-centric view of bioavailability and supports a microbiota-dependent framework in which functional outcomes are shaped by both substrate structure and microbial ecology [5].

This variability is particularly evident in the metabolism of dietary fibers and complex polysaccharides, where structural diversity leads to distinct fermentation profiles and metabolite production, including short-chain fatty acids with systemic regulatory roles [16,17]. Similarly, microbial cross-feeding and biosynthetic pathways contribute to vitamin metabolism and availability, further emphasizing the microbiota’s role in modulating nutrient functionality [18].

Importantly, the efficiency of microbial biotransformation appears to be constrained more by microbial functional capacity than by substrate availability alone, representing a key limitation in predicting dietary responses [11]. Consequently, taxonomic shifts alone fail to capture the functional impact of diet–microbiota interactions, highlighting the need for integrated multi-omics approaches to link accurately dietary inputs with metabolic and host outcomes [19].

### 2.1. Polyphenols and Gut Microbiota

Polyphenols comprise a structurally diverse class of plant secondary metabolites, including flavonoids, tannins, lignans, stilbenes, and phenolic acids, which are widely distributed in plant-derived foods [20]. Despite their well-established associations with reduced risk of cardiovascular, metabolic, and neurodegenerative diseases, their biological efficacy in humans remains paradoxically limited by poor bioavailability [21]. This apparent contradiction reflects a fundamental disconnect between chemical abundance and bio accessibility [13]. In contrast to readily absorbable nutrients, only a minor fraction of ingested polyphenols is absorbed in the small intestine, while the majority reaches the colon in structurally complex and often conjugated forms [13]. Host metabolism, primarily through phase I and II reactions, contributes mainly to conjugation and excretion rather than activation, thereby limiting the direct contribution of host pathways to their bioactivity [13]. In this regard, the gut microbiota emerges not simply as a complementary system but as a key contributing factor in shaping polyphenol functionality [14].

Once in the colon, polyphenols are subjected to a broad spectrum of microbial transformations that extend beyond the metabolic capabilities of the host [14]. These processes include deglycosylation, reduction, hydrolysis, demethylation, and ring cleavage, collectively facilitating the transformation of complex polyphenols into smaller, more bioaccessible metabolites [22]. Among these reactions, deglycosylation represents a critical initial step, as it removes sugar moieties and generates aglycones that are more susceptible to further microbial degradation [22]. Notably, this transformation highlights a key functional distinction: while host enzymes exhibit relatively narrow substrate specificity, gut microbial communities harbor a far more diverse enzymatic repertoire, enabling the processing of structurally complex dietary compounds [21]. However, this expanded metabolic capacity does not necessarily translate into uniform outcomes, as it remains highly dependent on microbial composition and enzymatic diversity [15].

Following initial transformation, gut microbes further degrade the flavonoid backbone through sequential enzymatic reactions that reshape the molecular structure into low-molecular-weight phenolic acids [14]. Although these metabolites generally exhibit improved absorption profiles compared to their parent compounds, it is essential to recognize that increased bioaccessibility does not inherently guarantee enhanced biological efficacy. Structural simplification may lead to the loss of specific functional groups responsible for certain bioactivities, while simultaneously generating metabolites with distinct or even divergent biological effects [23]. This underscores a pivotal nuance often eclipsed by the current research focus on absorption: the qualitative shifts in bioactivity that accompany metabolic degradation.

Interindividual variability becomes particularly evident in the metabolism of isoflavones, where microbiota composition dictates the production of specific metabolites such as (S)-equol [24]. While equol has been associated with enhanced estrogenic and cardiometabolic effects, only a subset of individuals harbor the microbial taxa required for its production [25]. This creates a clear dichotomy between “producers” and “non-producers,” illustrating that the health benefits of polyphenols cannot be generalized across populations. In this context, microbiota composition has been proposed to act as a metabolic gatekeeper, determining not only the extent but also the nature of polyphenol-derived bioactivity [15].

A similar complexity applies to non-flavonoid polyphenols, such as hydroxycinnamic acids, which undergo extensive microbial biotransformation into smaller phenolic derivatives with altered physicochemical and biological properties [22]. While these metabolites are often associated with antioxidant and anti-inflammatory effects, their activity depends on both their chemical structure and the microbial pathways involved in their generation [26]. This reinforces the notion that microbial metabolism may act as a biochemical filter, selectively shaping the pool of bioactive compounds that ultimately interact with the host.

More critically, polyphenols should not be interpreted as intrinsically bioactive entities, but rather as metabolic precursors whose physiological relevance is contingent upon microbial biotransformation efficiency [14]. The conversion of ellagitannins into urolithins exemplifies this principle, illustrating that host responses are often influenced largely by the metabolic competence of the resident microbiota to generate functionally active derivatives [27]. This perspective challenges reductionist interpretations that equate dietary polyphenol intake with predictable biological efficacy, emphasizing instead the decisive role of microbial functional capacity in determining downstream host effects.

Overall, the relationship between polyphenols and gut microbiota should not be interpreted as a simple linear pathway from ingestion to absorption [14]. Rather, it represents a dynamic and context-dependent process in which microbial metabolism is associated with reshaping both the bioavailability and functional activity of dietary compounds [14]. Importantly, variability in microbial composition introduces a layer of biological uncertainty that challenges conventional assumptions regarding the health benefits of polyphenol-rich diets. Addressing this complexity requires moving beyond a compound-centric perspective toward an integrated framework in which microbial functionality emerges as a central determinant of bioactivity [10].

### 2.2. Fatty Acids and Gut Microbiota

Building on the concept that gut microbiota acts as a key determinant of bioavailability for structurally complex dietary compounds, fatty acids introduce a fundamentally different mode of interaction [28,29]. Unlike polyphenols, whose bioavailability largely depends on direct microbial biotransformation, fatty acids influence the gut microbiota predominantly through indirect host-mediated mechanisms, particularly via bile acid metabolism and immune signaling pathways [30].

This distinction is critical. While microbial enzymes actively “unlock” polyphenols into absorbable metabolites [31], fatty acids, instead, may contribute to reshaping the intestinal environment that governs microbial functionality [32]. For instance, high-fat diets (HFDs) stimulate bile acid secretion, which exerts selective antimicrobial pressure and is associated with shifts in microbial composition [33]. Secondary bile acids such as deoxycholic acid further modulate dominant phyla, including Firmicutes and Bacteroidetes, suggesting that lipid-driven changes in bioavailability are mediated through ecological restructuring rather than direct chemical transformation [34].

However, interpreting these compositional shifts as direct proxies of functional outcomes remains problematic. Although high-fat diet (HFD)-induced alterations in gut microbiota have been associated with metabolic disorders and colorectal cancer, the relationship is not strictly linear, as similar compositional profiles may lead to divergent physiological responses [35]. In contrast, polyphenol metabolism often results in the generation of specific microbial-derived metabolites that can be more directly linked to biological activity [36], highlighting a fundamental difference in the predictability of host responses across dietary compound classes.

In contrast to saturated fats, unsaturated fatty acids-particularly n-3 polyunsaturated fatty acids (PUFAs)-are often associated with more favorable microbiota profiles and improved metabolic outcomes [37]. Yet, even in this case, the relationship between microbial modulation and host benefit remains inconsistent. While n-3 PUFAs can attenuate inflammatory signaling pathways such as TLR4 activation [28], clinical outcomes do not always correlate with observed microbial shifts, suggesting that microbiota composition alone cannot fully explain bioavailability-driven effects [28].

Importantly, the interaction between fatty acids and gut microbiota is bidirectional. Microorganisms can metabolize dietary lipids into bioactive intermediates such as conjugated linoleic acid (CLA), which exhibits anti-inflammatory properties [38]. However, this metabolic capacity is strain-specific and unevenly distributed across individuals, reinforcing the notion that microbial functionality—rather than mere presence—defines the bioavailability and physiological relevance of lipid-derived metabolites [39].

Further complexity arises when considering the source and processing of dietary fats. Diets rich in n-3 fatty acids (e.g., flaxseed or fish oil) tend to promote beneficial taxa such as Bifidobacterium, whereas saturated fat-rich diets often favor dysbiotic profiles [40]. Nevertheless, oxidized lipids generated during food processing may exert unpredictable or even detrimental effects on microbial composition, underscoring a critical limitation in extrapolating findings across dietary contexts [41].

Taken together, fatty acids illustrate a contrasting paradigm to polyphenols within the bioavailability framework [42]. Rather than serving primarily as substrates for microbial conversion, they may act as ecological modulators that reshape the microbial landscape governing metabolic outputs [32,43]. This distinction emphasizes that bioavailability should not be interpreted as a uniform concept across compound classes but rather as a context-dependent property shaped by the interplay between dietary chemistry, host physiology, and microbial functionality [15].

### 2.3. Polysaccharides and Gut Microbiota

Extending beyond polyphenols and fatty acids, plant-derived polysaccharides and dietary fibers represent a distinct class of bioactive compounds whose physiological effects are predominantly mediated through microbial fermentation rather than direct host absorption [15]. Unlike polyphenols, which require enzymatic transformation to generate absorbable metabolites, polysaccharides primarily function as substrates that support microbial metabolic activity, thereby exerting indirect yet profound effects on host physiology [17].

Upon reaching the colon, these structurally diverse carbohydrates undergo microbial fermentation, leading to the production of short-chain fatty acids (SCFAs), including acetate, propionate, and butyrate, which act as central mediators of host–microbiota interactions [16]. However, the extent and nature of these metabolic outputs are highly dependent on polysaccharide structure, including solubility, degree of polymerization, and chemical modifications, as well as the baseline microbial composition of the host [21].

For instance, resistant starch has been consistently associated with the enrichment of beneficial taxa such as *Faecalibacterium prausnitzii*, alongside reductions in opportunistic pathogens, suggesting a selective modulatory effect on gut microbial ecology [17]. Similarly, soluble fibers from cereal sources have been shown to increase *Bifidobacterium* and *Lactobacillus* abundance while enhancing SCFA production, reflecting elevated fermentative activity [15]. In contrast, some fiber interventions induce minimal changes in overall microbial composition despite significantly altering metabolic outputs, highlighting a key limitation of relying solely on taxonomic descriptors [44].

This discrepancy between compositional and functional responses underscores a critical distinction between dietary fibers and other bioactives produced by gut microbiota [45]. While polyphenol metabolism often results in discrete and chemically identifiable metabolites [46], fiber fermentation produces a relatively conserved set of end products, yet with highly variable quantitative outputs depending on microbial ecology [19]. Consequently, functional readouts, rather than compositional shifts alone, provide a more accurate representation of microbiota-mediated effects [19].

Importantly, interindividual variability remains a defining feature of polysaccharide–microbiota interactions [19]. Differential responses to structurally similar substrates- such as feruloylated oligo- and polysaccharides-have been linked to variations in microbial taxa, including *Blautia* and *Akkermansia*, as well as differences in SCFA production [15]. These findings reinforce the concept that microbial functionality, rather than substrate availability alone, may govern metabolic outcome.

Beyond metabolic regulation, polysaccharides also influence host immune responses through microbiota-dependent mechanisms [47]. Experimental evidence indicates that microbiota modulation by specific polysaccharides has been associated with enhanced therapeutic responses, including improved efficacy of immune-targeted interventions, further supporting a role for microbial composition and function in shaping host physiology [47].

Despite these advances, key gaps remain. The relationship between polysaccharide structure and microbial functionality is not fully resolved, and the impact of food processing on fermentability and metabolic outcomes remains insufficiently explored [48]. Addressing these limitations will be essential for translating dietary fiber research into targeted and personalized nutritional strategies.

Table 1 presents an overview of dietary bioactive compounds and their gut microbiota interactions. This highlights microbial-mediated mechanisms (e.g., SCFA production, cross-feeding) and functional host outcomes across various dietary classes, while identifying current limitations such as over-reliance on taxonomic shifts.

Table 1 is a functional overview of dietary–microbiota interactions across distinct evidence hierarchies. Representative microbial taxa and metabolites are presented as illustrative examples of dominant mechanistic pathways rather than exhaustive determinants. Evidence strength is classified according to the predominant experimental framework supporting each interaction (mechanistic, preclinical, observational human, or clinical intervention), thereby distinguishing mechanistic plausibility from validated translational relevance.

**Figure 1:**
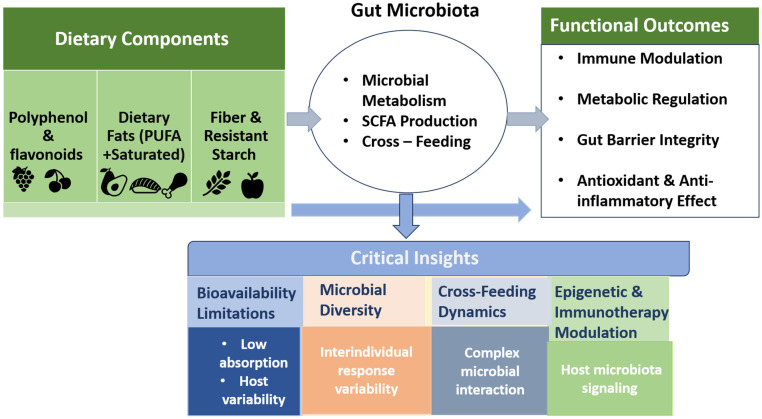
Diet-Microbiota Interactions and Functional Outcomes [Drawn by first author].

The integrative overview of the dietary–microbiota metabolic network highlights that functional host outcomes emerge through dynamic bidirectional interactions involving microbial biotransformation, cross-feeding relationships, and host-responsive signaling pathways. Rather than representing a linear diet-to-effect cascade, this framework emphasizes a context-dependent metabolic architecture in which identical dietary substrates may yield divergent physiological outcomes depending on microbial composition, enzymatic functionality, and host-specific regulatory capacity. Importantly, Figure 1 reflects the uneven translational maturity of current evidence, with several mechanistic pathways strongly supported by preclinical models but still requiring validation in large-scale human studies. This systems-level variability provides a mechanistic basis for the substantial inter-individual heterogeneity consistently observed across clinical and nutritional intervention studies.

The figure illustrates the principal microbiota-mediated transformation routes of major dietary bioactive classes (polyphenols, dietary lipids, and polysaccharides), highlighting representative microbial taxa, key metabolites (e.g., SCFAs, urolithins, bile acid derivatives), primary host molecular targets, and associated physiological outcomes. Pathway representation reflects current evidence hierarchy across mechanistic, preclinical, and human studies, emphasizing translational limitations and context-dependent variability.

Collectively, these interactions demonstrate that microbiota-mediated metabolism is compound-specific and context-dependent. While polyphenols require microbial activation, fatty acids primarily influence composition, and polysaccharides serve as fermentable substrates [20]. This functional heterogeneity underscores the need to transition from generalized descriptions toward a mechanistic understanding of specific microbial transformation pathways [54,55]. Accordingly, the following section focuses on the biochemical routes through which gut microbiota convert distinct classes of bioactive compounds into functionally active metabolites.

## 3. Pathways of Bioactive Transformation

Microbiota-mediated metabolism of dietary bioactive compounds should not be interpreted as a passive or purely degradative process but rather as a highly coordinated biochemical system that reshapes the structural and functional properties of dietary molecules [5]. While previous sections have emphasized the variability and microbiota-dependence of bioaccessibility, a mechanistic understanding of these metabolic shifts requires a closer examination of the enzymatic pathways and metabolic networks involved [15].

At the molecular level, gut microbial communities catalyze a wide range of biochemical reactions, including hydrolysis, reduction, dehydroxylation, and ring cleavage, that collectively convert structurally complex dietary substrates into smaller, chemically distinct metabolites [14,56]. Importantly, these biochemical processes are not uniform across compound classes; instead, they reflect substrate-specific metabolic routes governed by microbial enzymatic repertoires and ecological interactions [22].

This functional specialization introduces an additional layer of complexity, as microbial metabolism acts not merely as a converter of dietary inputs but as a selective biochemical filter that determines the composition of the metabolite pool reaching the host [23]. Consequently, understanding these transformation pathways is essential for linking dietary bioactives, particularly those derived from fermented food, to their downstream biological effects [57].

Accordingly, the following sections focus on the major microbiota-mediated transformation routes of key dietary bioactives, with particular emphasis on polyphenols, ellagitannins, and fermentable polysaccharides, highlighting both shared mechanisms and compound-specific metabolic signatures. Within this context, polyphenols represent one of the most extensively studied yet metabolically complex classes of dietary bioactives, making them an ideal model for understanding microbiota-driven biotransformation processes.

### 3.1. Polyphenol Transformation Pathways

Polyphenols constitute a structurally diverse group of plant secondary metabolites, including flavonoids, tannins, lignans, stilbenes, and phenolic acids, widely present in plant-derived materials, including various plant-based matrices, which are a rich source of antioxidant activities [58,59]. Despite their well-documented associations with reduced risk of cardiometabolic and neurodegenerative diseases, their biological efficacy remains constrained by inherently low bioavailability [13,60]. This apparent paradox reflects a critical limitation: the majority of ingested polyphenols reach the colon in complex forms, where host metabolism contributes primarily to conjugation rather than activation [14,21]. Consequently, the gut microbiota emerges as the principal determinant of polyphenol functionality [22,46].

Within the colon, polyphenols undergo extensive microbial biotransformation, including deglycosylation and ring cleavage, yielding smaller and more bioaccessible metabolites [27,46]. This process highlights a fundamental distinction: while host enzymes exhibit narrow specificity, the gut microbiota possesses a broader enzymatic repertoire (such as CAZymes) capable of processing complex compounds [61]. However, this metabolic flexibility introduces variability, as these transformations are highly dependent on individual microbial composition [56,62].

Importantly, enhanced bioaccessibility does not necessarily equate to increased biological efficacy, as microbial degradation may generate metabolites with distinct or context-dependent functions [63,64]. Inter-individual variability further complicates this, as seen in the microbial conversion of isoflavones into (S)-equol, produced only in a subset of individuals harboring specific microbial taxa [24,25]. This “producer vs. non-producer” dichotomy illustrates that microbiota composition acts as a metabolic gatekeeper [65,66]. Collectively, these observations support a shift toward a microbiota-centered framework, where microbial functionality defines biological outcomes [9,10].

Evidence remains predominantly derived from in vitro biotransformation models and preclinical systems, whereas controlled human intervention studies capable of validating these mechanistic predictions remain comparatively scarce [3,62].

### 3.2. Transformation of Ellagitannins to Urolithin

Building upon microbiota-driven polyphenol metabolism, the conversion of ellagitannins into urolithins represents a prototypical example of microbiota-dependent bioactivation [36,67]. Due to their complex polymeric structure, ellagitannins—abundant in agri-food by-products such as peels and pomace—exhibit minimal direct absorption, rendering their biological activity largely contingent on microbiota-mediated transformation [50,52]. In the colon, they are hydrolyzed to ellagic acid, which is subsequently converted into urolithins through sequential microbial reactions, including reduction and dehydroxylation [36,37].

However, this transformation is highly dependent on microbial composition and is exhibits significant inter-individual variability; specific microbiota configurations are capable of producing urolithins, giving rise to distinct metabotypes that influence physiological outcomes [50,52]. This inter-individual variability challenges the notion of a linear diet–metabolite–effect relationship and underscores the limitations of extrapolating in vitro findings to in vivo systems [36,37].

Notably, the current evidence supporting urolithin-associated biological effects remains uneven across experimental systems [3]. While in vitro and animal studies consistently demonstrate mechanistic links between urolithin exposure and epigenetic modulation, translation into human physiology remains comparatively limited [9], with most available evidence deriving from observational associations or small-scale intervention studies rather than large randomized clinical trials [67]. This discrepancy highlights an important translational gap and cautions against overgeneralizing mechanistic findings across evidence hierarchies [3,9].

A major unresolved limitation lies in the incomplete characterization of the enzymatic and genetic basis underlying urolithin biosynthesis. Although the pathway involves defined chemical reactions, the specific enzymes and their genomic context remain poorly mapped [53,68]. This mechanistic gap highlights a broader limitation in microbiota research, where functional pathways are often inferred rather than experimentally validated [17,68]. The observation that phylogenetically diverse bacteria can contribute to urolithin production further suggests either convergent evolution or conserved functional modules [15].

Importantly, microbial composition alone does not reliably predict metabolic function. Changes in the abundance of taxa such as Bacteroides or Faecalibacterium do not consistently correlate with urolithin production [54,55]. This emphasizes the need for function-oriented frameworks that account for metabolic redundancy [9,15]. From a systems perspective, urolithin production emerges from coordinated metabolic interactions within microbial consortia [55,68].

Despite advances, an integrative mechanistic model remains lacking [27,56]. This gap underscores the need for multi-omics integration [17]. Collectively, urolithin production is an emergent property of a context-dependent microbial ecosystem [1,36]. This reinforces the need for precision nutrition strategies [56,69]. Notably, this paradigm extends beyond polyphenols, as seen in the microbial fermentation of dietary fibers into short-chain fatty acids (SCFAs) [70,71]. However, unlike SCFA production, urolithin biosynthesis is highly specialized and variable [36,69].

### 3.3. Dietary Fiber–Microbiota Interactions Beyond SCFAs

Dietary fibers exert profound effects on host physiology that extend far beyond their role as mere substrates for short-chain fatty acid (SCFA) production [72]. While SCFAs such as acetate, propionate, and butyrate are widely recognized for their epigenetic and metabolic signaling functions, a growing body of evidence highlights additional microbial-derived metabolites—including bile acids (BAs) and neuroactive compounds-as critical mediators of host cellular pathways [55,72,73]. These metabolites operate within an interconnected metabolic network shaped by fiber type, solubility, microbial composition, and host-specific factors, reinforcing the concept of the gut microbiota as a dynamic and context-dependent metabolic system [19,54]. Importantly, this expanded view aligns with recent systems-level frameworks that position the gut microbiota as an active regulator of host epigenomic plasticity rather than a passive metabolic compartment [3,11]. This perspective is particularly relevant in the context of plants and grain-based nutrition, where fiber-rich matrices act not only as substrates but also as modulators of microbiota-derived signaling networks [57,58].

The functional consequences of this network are multifaceted. SCFAs directly modulate chromatin accessibility through histone deacetylase (HDAC) inhibition and activate G protein-coupled receptors to regulate systemic metabolic homeostasis [16,74].

While this epigenetic potential is extensively documented, its direct translation into consistent human clinical outcomes remains less definitive. This translational inconsistency likely reflects the rapid metabolic turnover of SCFAs, their compartment-specific bioavailability, and pronounced interindividual differences in baseline microbial composition, collectively suggesting that the effective “epigenetic dosage” reaching target tissues is substantially more complex than simplified fermentation-output models often assume [74]. This observation challenges reductionist interpretations that equate increased SCFA production with predictable host benefit, underscoring instead the necessity of context-sensitive functional assessment that integrates evidence from preclinical, observational, and clinical human studies [19,62].

In parallel, secondary bile acids (2°BAs) and microbially conjugated bile acids (MCBAs) function as ligands for nuclear and membrane-bound receptors, thereby influencing energy metabolism, immune responses, and epigenetic regulation [43,72]. Moreover, gut-derived neuroactive compounds-including γ-aminobutyric acid (GABA), serotonin, and dopamine-interact with enterochromaffin cells and neural circuits to modulate gut–brain communication and host physiology [75,76].

To clarify these mechanistic pathways, the following subsections detail SCFA-mediated epigenetic and metabolic signaling, followed by the regulatory roles of non-SCFA microbial metabolites, including bile acids and neuroactive compounds [72,77]. Such a structure enables a clearer comparison of mechanistic pathways, highlights fiber-specific effects, and supports the development of personalized dietary strategies grounded in host–microbiota interactions [62,78].

While SCFAs form a foundational axis linking dietary fibers to host epigenetic regulation and metabolic signaling, importantly, this metabolic framework extends beyond fiber fermentation alone, as polyphenols derived from agri-food by-products undergo extensive microbial biotransformation into bioactive metabolites (e.g., phenolic acids and urolithins) that converge on similar epigenetic and signaling pathways, thereby reinforcing the integrative nature of the fiber–polyphenol–microbiota axis [67,79].

#### 3.3.1. Epigenetic and Cellular Signaling—SCFAs

The fermentation of dietary fibers by the gut microbiota represents the primary mechanistic interface between diet and host cellular epigenetics [3]. In the colon, fibers escaping upper digestion are hydrolyzed by microbial Carbohydrate-Active Enzymes (CAZymes), including glycoside hydrolases and polysaccharide lyases, which orchestrate the breakdown of complex structural matrices [61]. This process yields SCFAs—primarily acetate, propionate, and butyrate—which function as potent endogenous inhibitors of Histone Deacetylases (HDACs) [80].

By inhibiting HDACs, SCFAs promote histone acetylation and an open chromatin configuration (euchromatin), thereby enhancing transcription factor accessibility to promoters governing anti-inflammatory pathways and epithelial barrier integrity [81,82]. Notably, this epigenetic modulation operates in a context-dependent manner; concentration gradients and cellular metabolic states determine whether butyrate acts as a signaling regulator or an oxidative energy substrate [16]. Beyond nuclear effects, SCFAs engage membrane-bound G protein-coupled receptors, specifically GPR41 and GPR43, to regulate systemic metabolic homeostasis and immune responses, effectively translating microbial metabolic output into functional host phenotypes [83,84].

#### 3.3.2. Bile Acids and Neuroactive Compounds—Beyond SCFAs

The scope of microbiota-dependent cellular modulation extends to the transformation of primary bile acids (BAs) into secondary BAs (2°BAs), which act as high-affinity ligands for the TGR5 receptor and the nuclear Farnesoid X Receptor (FXR) [72]. Activation of these pathways recruits chromatin-modifying enzymes, linking microbial BA metabolism directly to host transcriptional regulation and metabolic health [78].

Fiber solubility and structure (Table 2) play decisive roles in shaping these outcomes; for instance, soluble fibers like inulin have been shown to promote 2°BA production and modulate type 2 inflammation, whereas insoluble fibers like cellulose typically exert minimal metabolic shifts [85,86]. In parallel, dietary fibers modulate the production of neuroactive compounds, including GABA and serotonin, predominantly by fiber-degrading taxa such as Bifidobacterium and Akkermansia [35,76]. However, the evidence supporting these neuroactive effects remains predominantly preclinical, with limited confirmation in controlled human intervention studies, reinforcing the need for cautious interpretation when extrapolating mechanistic findings to clinical outcomes [5,9].

Table 2 summarizes representative fiber-specific microbiota interactions across distinct evidence hierarchies. Evidence strength varies considerably between fiber classes, with mechanistic support deriving predominantly from animal and controlled intervention studies, whereas large-scale human validation remains limited. The reported microbial and host responses should therefore be interpreted as context-dependent rather than universally generalizable.

These comparisons illustrate that soluble and highly fermentable fibers preferentially modulate metabolically active microbial consortia capable of generating signaling metabolites with epigenetic relevance, whereas poorly fermentable fibers exert comparatively limited regulatory effects. Importantly, the translational strength of these observations remains uneven across evidence systems, with many mechanistic insights still relying on preclinical models. This evidence asymmetry reinforces the need for standardized multi-omics human studies capable of resolving host-specific response variability and establishing clinically actionable fiber–microbiota signatures [9,19].

In conclusion, dietary fibers orchestrate a complex microbial metabolic network extending beyond SCFA production [11]. The interplay of fiber type, solubility, and host–microbiota context shapes BA diversity, MCBA formation, and neuroactive metabolite production, thereby modulating intracellular signaling and nuclear accessibility [72]. Integrating single-cell transcriptomics and metabolomics in future studies will be crucial to decipher the precise mechanisms by which these fiber-derived metabolites influence cellular fate and epigenetic plasticity, providing a mechanistic basis for personalized nutrition and immune-metabolic health [19]. From a translational perspective, these findings position dietary fibers derived from plant-based nutrition as promising tools in the form of prebiotics for precision nutrition strategies aimed at modulating host epigenetic and metabolic states through microbiota engineering [4,87].

## 4. Epigenetic and Systemic Modulators

The host–microbiota relationship extends beyond simple coexistence, representing a dynamic biochemical and regulatory interface [3]. Microbiota-derived metabolites (MDMs) are increasingly recognized as pivotal epigenetic modulators, capable of influencing host gene expression and chromatin architecture and cellular signaling pathways [10]. Unlike classical nutrients, which primarily serve metabolic or energetic roles, MDMs act as bioactive signaling molecules, bridging microbial activity with host transcriptional programs [9].

Short-chain fatty acids (SCFAs), generated through microbial fermentation of dietary fibers, have emerged as prototypical MDMs with broad epigenetic effects, including histone acetylation, DNA methylation, and modulation of chromatin accessibility [16,74]. These metabolites demonstrate tissue-specific and dose-dependent actions, influencing not only local intestinal epithelial cells but also systemic immune and metabolic responses [8].

Beyond SCFAs, other microbiota-derived metabolites—such as polyphenol-derived compounds and tryptophan catabolites—exert targeted epigenetic effects on host barrier function [23,88]. Similarly, bile acids and branched-chain amino acids (BCAAs) have been identified as systemic modulators of chromatin accessibility [30,89]. These metabolites influence immune function and metabolic homeostasis through mechanisms that are highly context-sensitive [78]. Furthermore, cellular differentiation is orchestrated by these molecules in response to host genetics and microbiota composition [3,90].

Collectively, this evidence positions MDMs as central mediators linking gut microbial activity with host transcriptional and epigenetic programming [9,18]. Such interactions highlight their potential as precise targets for nutritional and therapeutic interventions [10]. This section critically examines the major classes of microbial metabolites and their mechanisms of epigenetic regulation. Finally, the functional consequences on host physiology are analyzed, emphasizing mechanistic diversity and translational relevance.

### 4.1. Multifaceted Epigenetic and Systemic Modulators—SCFAs

Microbial-derived short-chain fatty acids (SCFAs), including acetate, propionate, and butyrate, arise from the fermentation of dietary fibers by commensal gut bacteria such as *Bacteroides* spp., *Clostridium* clusters IV/XIVa, and *Faecalibacterium prausnitzii* [36,74]. Beyond their role as simple energy substrates, SCFAs act as bioactive metabolites bridging the gut microbiota to host immunity, metabolism, and epigenetic regulation [11]. Distinct from traditional dietary components, SCFAs can influence chromatin structure, histone modification, and gene expression patterns, suggesting their participation in long-range systemic signaling rather than purely local metabolic functions [91]. Future studies should delineate the combinatorial effects of SCFAs with other microbial metabolites on tissue-specific epigenomes.

SCFAs exert part of their effects via G-protein-coupled receptors (GPCRs), including GPR41, GPR43, and GPR109A, expressed in immune cells, adipose tissue, liver, and even the central nervous system [84]. Propionate activation of GPR43, for instance, directs T cell differentiation and mitigates systemic inflammation, highlighting a critical role in cardiovascular protection [83]. Acetate, while less potent locally, modulates hypothalamic circuits affecting energy homeostasis and neuroimmune signaling [92]. In the context of human disease, these pathways appear to contribute to immune–metabolic regulation; however, their clinical relevance remains dependent on context and is not yet fully established across large-scale human studies [82].

Beyond receptor-mediated pathways, SCFAs, particularly butyrate, act as histone deacetylase (HDAC) inhibitors, altering chromatin accessibility and transcriptional programs in epithelial, immune, and even neuronal cells [80]. Propionate and acetate share HDAC-inhibitory activity but with reduced potency and tissue specificity, which may explain differential systemic outcomes [92]. Importantly, these epigenetic effects are context-dependent: butyrate can simultaneously activate p300-mediated histone acetylation while inhibiting HDACs, reflecting complex, concentration- and tissue-specific responses [93]. Despite strong mechanistic evidence in animal models, translation to humans remains limited due to species-specific microbiota composition, tissue-specific SCFA concentrations, and inter-individual variability, highlighting the need for precision microbiota-based interventions.

SCFAs exhibit overlapping yet distinct roles in metabolism, immunity, and cancer regulation (Table 3).

Table 3 integrates current mechanistic evidence linking SCFAs to host epigenetic regulation across multiple experimental systems. Although butyrate-mediated HDAC inhibition is supported by substantial mechanistic evidence, translational consistency across human studies remains limited by metabolite compartmentalization, rapid turnover kinetics, and baseline microbiota-dependent variability.

The biological significance of SCFAs within the intestinal microenvironment is defined by a sophisticated interplay between metabolic flux and chromatin remodeling [10,11]. Unlike systemic signaling molecules, SCFAs, specifically butyrate, operate through a “Metabo-Epigenetic” framework [74]. In healthy, differentiated colonocytes, the rapid sequestration of butyrate into the mitochondria for beta-oxidation serves a dual cytological purpose: it fuels oxidative phosphorylation (OXPHOS) to maintain mucosal hypoxia and simultaneously limits the nuclear translocation of butyrate, thereby preventing excessive global HDAC inhibition [29,77]. However, much of this mechanistic framework derives from in vitro and animal models, and direct extrapolation to human physiology remains constrained by substantial variability in luminal concentrations, epithelial uptake efficiency, and microbiota composition. This translational uncertainty highlights the need for quantitatively resolved human studies capable of linking local SCFA dynamics to tissue-specific epigenetic responses.

Furthermore, the discovery of Histone Crotonylation (Kcr) as a butyrate-dependent modification introduces a new layer of regulatory specificity [10,74]. This non-canonical modification is chemically distinct from acetylation and appears to specifically mark active pro-metabolic gene loci, suggesting that the microbiota directly provides the carbon backbone for structural chromatin remodeling [11,80].

The transition from the localized, potent effects of butyrate to the systemic, receptor-mediated effects of acetate and propionate reflects a calculated trade-off in cellular signaling [16,36]. While butyrate is the primary driver of Intestinal Epithelial Cell (IEC) homeostasis and apoptosis in malignant cytology, acetate serves as a vital bridge to systemic immune fitness, crossing the blood–brain barrier to modulate neuro-cytological environments [78,92]. This divergence underscores the necessity of analyzing SCFAs not as generic metabolites, but as context-dependent epigenetic ligands whose functional outcome is dictated by the metabolic state and lineage of the host cell [91,93].

### 4.2. Regulatory Potential of Polyphenol-Derived Metabolites

Polyphenol-derived metabolites represent a distinct class of microbiota-dependent signaling molecules whose regulatory potential extends beyond simple antioxidant activity to encompass precise modulation of host epigenetic and cellular pathways [3]. In contrast to short-chain fatty acids (SCFAs), which primarily act as broad-spectrum chromatin modulators through histone deacetylase (HDAC) inhibition, polyphenol-derived metabolites exhibit a higher degree of molecular specificity, targeting defined enzymes, transcription factors, and signaling cascades in a context-dependent manner [14].

This functional divergence originates from their reliance on microbial biotransformation. Given that only a minor fraction of dietary polyphenols is absorbed in the small intestine, the majority undergoes extensive enzymatic conversion in the colon, yielding structurally simplified metabolites such as phenolic acids, γ-valerolactones, and urolithins with enhanced bioavailability and systemic reach [67]. Unlike SCFAs, whose effects are largely concentration-dependent and localized within the gut, these metabolites frequently circulate as conjugated forms, enabling prolonged interaction with peripheral tissues and distant epigenetic targets in experimental and preclinical systems [12].

Urolithin A, for instance, activates SIRT1-dependent pathways, promoting mitophagy and mitochondrial quality control while simultaneously influencing chromatin stability and transcriptional regulation in cellular and animal models [27]. Similarly, flavanol-derived phenolic acids modulate redox-sensitive transcription factors and inflammatory signaling pathways, whereas resveratrol-derived metabolites regulate endothelial and hepatic gene expression through sirtuin activation and epigenetic remodeling [79].

Importantly, these mechanisms are not independent of microbial ecology. Polyphenols simultaneously reshape the gut microbiota by selectively enriching taxa such as *Faecalibacterium prausnitzii* and *Akkermansia muciniphila*, thereby establishing a metabolic feedback loop that enhances SCFA production, particularly butyrate [16,17]. Thus, rather than acting in isolation, polyphenol-derived metabolites and SCFAs converge mechanistically on shared immune and metabolic endpoints through complementary routes [11].

A critical limitation, however, lies in the pronounced interindividual variability in polyphenol metabolism. The formation of key metabolites such as urolithins or equol is restricted to specific microbial “metabotypes,” reflecting differences in enzymatic capacity rather than substrate availability [56,66].

Metabolic data (Table 4) reveal that unlike non-selective HDAC inhibition by SCFAs, polyphenol-derived MDMs act as specific ligands for mitochondrial and nuclear regulatory pathways. A key example is the Urolithin-mediated ‘mitochondrial-epigenetic’ axis, where SIRT1 activation links mitophagy to the transcriptional silencing of pro-inflammatory cytokines like IL-6 and TNF-α.

Table 4 summarizes representative microbiota-derived polyphenol metabolites across distinct evidence hierarchies. Although multiple mechanistic pathways have been identified, translational confidence varies considerably across metabolite classes, with much of the available evidence remaining preclinical or observational. Consequently, these effects should be interpreted as microbiota-contingent and context-dependent rather than universally reproducible.

The diversity of microbial metabolites generated from structurally distinct polyphenols illustrates that the gut microbiota functions not merely as a degradative system, but as a selective biochemical bioreactor capable of producing epigenetically active molecules distinct from their dietary precursors [3]. However, the translational relevance of these pathways remains uneven across metabolite classes. While mechanistic studies consistently demonstrate molecular interactions ranging from ERβ engagement by equol to Nrf2-mediated antioxidant signaling by phenolic acids, direct validation in human physiological systems remains comparatively limited for many pathways. This discrepancy highlights an important evidence hierarchy that cautions against extrapolating preclinical findings into generalized nutritional recommendations. Consequently, advancing toward precision nutrigenomics will require function-oriented frameworks capable of integrating microbial metabolic competence, host responsiveness, and evidence-level stratification when predicting therapeutic outcomes [9,19].

## 5. Cross-Kingdom Regulatory Networks: Epigenetic Programming

Epigenetic regulation represents the functional interface through which microbial metabolites, introduced in previous Section, translate environmental and dietary inputs into stable yet reversible changes in host gene expression [3,11]. While short-chain fatty acids (SCFAs) and polyphenol-derived metabolites differ in their biochemical origin and specificity, they converge mechanistically at the level of chromatin remodeling, DNA methylation, and non-coding RNA regulation [14]. This convergence defines a dynamic “microbiota–epigenome axis” that governs cellular phenotype, metabolic homeostasis, and disease susceptibility [91]. Importantly, this axis is not static; it is continuously reshaped by dietary exposures, microbial composition, and host metabolic state, positioning epigenetic regulation as a central mediator linking microbiota-derived metabolites to systemic physiological outcomes [91]. Moreover, the magnitude and specificity of these epigenetic modifications are highly dependent on the host’s unique microbial composition, linking responses to individual metabotypes [12,67].

### 5.1. DNA Methylation and Gene Expression Control

Metabolites in gut influence DNA methylation patterns by modulating the availability of methyl donors and regulating DNA methyltransferase (DNMT) activity [97]. These effects are highly context-dependent and reflect the resident microbial landscape [23]. Variations in microbiota profiles, particularly shifts in dominant phyla, have been associated with differential methylation of genes involved in lipid metabolism, insulin signaling, and adipogenesis [93]. Notably, the microbial provision of methyl donors is mediated by folate (Vitamin B9) and Vitamin B12, synthesized by bacteria such as *Bifidobacterium* and *Lactobacillus*, which feed into the one-carbon metabolism cycle to generate *S*-adenosylmethionine (SAM) the universal methyl donor [97]. This metabolite directly influences DNMT modulation, thereby regulating DNA methylation at promoters of genes involved in lipid uptake, glucose homeostasis, and inflammatory pathways [10,74,98].

The regulation of host metabolism extends beyond direct signaling, involving epigenetic control of key metabolic genes such as those governing leptin sensitivity and glucose homeostasis [80,88]. This suggests that dysbiosis-driven alterations in methylation landscapes may contribute to persistent metabolic dysfunction, reinforcing the concept that microbiota composition can imprint long-term metabolic phenotypes through epigenetic memory [12]. Importantly, these methylation patterns vary significantly among individuals depending on the unique microbial composition and functional capacity of their gut microbiota [94].

### 5.2. Histone Modifications and Chromatin Remodeling

At the level of chromatin structure, microbial metabolites act as modulators of histone acetylation and deacetylation, thereby controlling transcriptional accessibility [11]. For instance, HDAC inhibition by butyrate has been shown to upregulate the expression of CD36 in intestinal epithelial cells, thereby enhancing fatty acid uptake and directly impacting lipid absorption and systemic metabolic flux [29,77]. Conversely, polyphenol-derived microbial metabolites exert a more selective modulation, potentially activating histone acetyltransferases (HATs) or influencing methyltransferases to create complex chromatin states that fine-tune gene expression in a tissue-dependent manner [23,78].

This regulation is evident in metabolic tissues, where microbiota-dependent activation of HDACs influences lipid storage through transcriptional control of transporters [10]. Importantly, histone modifications are not uniformly beneficial; their effects depend on tissue context and metabolic state, highlighting a dual regulatory role in both physiological adaptation and disease progression [11]. Furthermore, emerging evidence indicates that microbial metabolites can simultaneously activate HATs and inhibit HDACs, creating complex and sometimes opposing chromatin states that fine-tune gene expression [3].

### 5.3. Non-Coding RNAs and Post-Transcriptional Regulation

A critical layer of microbiota-driven epigenetic regulation involves non-coding RNAs, particularly microRNAs (miRNAs), which modulate gene expression post-transcriptionally [10,27]. The gut microbiota has been shown to regulate host miRNA expression profiles, thereby influencing pathways related to glucose metabolism, adipocyte differentiation, and inflammatory signaling [9,89]. Microbial metabolites-including lipopolysaccharide-derived signals and secondary fermentation products-act as upstream regulators of miRNA networks, linking microbial activity to host metabolic control. The dynamic nature of miRNA regulation allows rapid adaptation to dietary and microbial changes, complementing the slower and more stable DNA methylation patterns [23,78].

### 5.4. Disease-Relevant Epigenetic Reprogramming

The epigenetic effects of microbial metabolites become particularly evident in disease contexts characterized by immune dysregulation and metabolic imbalance [77]. In inflammatory bowel disease, microbial-derived compounds have been associated with modulation of chromatin accessibility at inflammatory gene loci, with mechanistic studies suggesting possible involvement in the attenuation of NF-κB signaling and inflammasome activation through combined signaling and epigenetic mechanism [93,94]. These effects may contribute to the maintenance of epithelial barrier integrity and immune homeostasis through the regulation of epithelial tight-junction integrity and inflammatory transcriptional responses [7,16].

Similarly, in colorectal cancer, microbial metabolites have been implicated in pathways associated with tumor progression through epigenetic regulation of oncogenic pathways, including Wnt/β-catenin and JAK/STAT signaling, which may influence proliferative and apoptotic responses depending on metabolite concentration, tissue context, and host–microbiota composition [80,99]. This context dependency is particularly relevant to the butyrate paradox, whereby butyrate may exert anti-proliferative effects in transformed colonocytes while supporting oxidative metabolism in healthy epithelial cells [74,80,94].

In functional disorders such as IBS, microbiota-derived metabolites extend their influence to the gut–brain axis, where epigenetic modulation of neurotransmitter-related genes contributes to altered visceral sensitivity and neuroimmune signaling [78]. Emerging evidence further suggests that these interactions involve bidirectional communication among microbial metabolites, enteroendocrine signaling, and neural circuitry, although causal relationships remain incompletely resolved in clinical settings [9,78].

A deeper mechanistic layer underlying disease-specific microbiota–epigenome interactions involves the integration of metabolite-driven signaling with host epigenetic memory and immune cell training [7,9]. Microbial metabolites do not act as isolated effectors, but instead shape long-term transcriptional responsiveness through sustained modulation of chromatin accessibility and DNA methylation patterns, thereby contributing to persistent immune reprogramming across disease states [10]. This is particularly relevant in chronic inflammatory and metabolic disorders, where repeated exposure to microbial metabolites can reinforce maladaptive or protective epigenetic states depending on host context and microbial composition [5]. Moreover, inter-individual variability in microbiota functional capacity introduces an additional regulatory dimension, whereby identical metabolites may trigger divergent epigenetic outputs across patients [19]. This variability is further amplified by host genetic background and early-life microbial colonization, suggesting that disease phenotypes emerge from layered interactions between microbial metabolism, epigenetic plasticity, and immune system priming rather than single-pathway regulation [7].

These observations support a balanced interpretation of disease-related mechanistic evidence and emphasize the need for evidence-stratified translation from preclinical systems to human disease contexts.

### 5.5. Dietary Modulation of the Microbiota–Epigenome Axis

Dietary polyphenols from agri-food by-products represent a critical upstream driver of microbiota-mediated epigenetic regulation. Rather than acting directly, their effects are largely mediated through microbial biotransformation into bioactive metabolites that influence host chromatin dynamics [11,79]. Polyphenol-rich sources-such as pomegranate, berries, and cocoa-selectively enrich beneficial taxa (e.g., *Akkermansia*, *Bifidobacterium*), which in turn enhance the production of SCFAs and phenolic metabolites [12,88]. For instance, ellagitannin-rich by-products promote the generation of urolithins, linked to SIRT1 activation and mitochondrial quality control, whereas anthocyanin-rich residues amplify histone acetylation pathways through enhanced SCFA production [27,67].

Taken together, these findings establish the microbiota–epigenome axis as a central mechanistic framework linking diet, microbial metabolism, and host physiology [11]. Microbial metabolites function not merely as signaling intermediates but as active regulators of chromatin architecture and gene expression. This cross-kingdom dialog suggests that the host genome is not a rigid blueprint, but a dynamic substrate programmed by microbial metabolites, which integrates individual microbiota composition (metabotypes) into personalized epigenetic responses [3].

**Figure 2:**
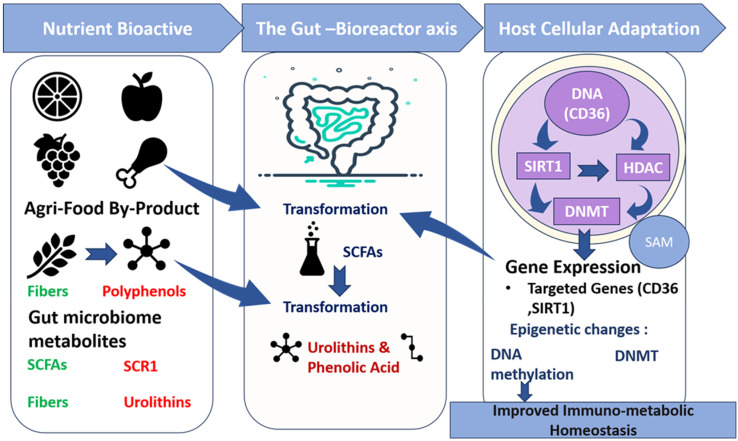
Molecular Orchestration of Epigenetic Regulation by Microbial Metabolites. [Drawn by first author].

These molecular interactions are summarized in Figure 2, which provides an integrative mechanistic framework linking fiber- and polyphenol-derived microbial metabolites to host epigenetic regulation. Unlike a purely descriptive schematic, this model emphasizes a hierarchical signaling cascade that begins with microbial metabolic activity in the gut lumen and culminates in the modulation of chromatin architecture within host target cells. The figure highlights key epigenetic regulators, including histone deacetylases (HDACs), DNA methyltransferases (DNMTs), and sirtuin 1 (SIRT1), which collectively integrate metabolic cues into transcriptional reprogramming. Importantly, this pathway representation reflects the current evidence landscape, where strong mechanistic support exists for SCFA-mediated HDAC inhibition, while DNMT and SIRT1-related effects of polyphenol metabolites remain comparatively context-dependent and less uniformly validated in human studies. This distinction underscores the translational heterogeneity of microbiota-derived epigenetic regulation and the need for evidence-stratified interpretation.

The figure illustrates the multi-layered signaling cascade through which gut microbial metabolites (e.g., short-chain fatty acids, phenolic acids, and urolithin derivatives) regulate host epigenetic machinery. Key regulatory nodes include HDAC inhibition, DNMT modulation, and SIRT1 activation, leading to chromatin remodeling and downstream transcriptional reprogramming associated with immune-metabolic homeostasis. The schematic integrates mechanistic insights across in vitro, animal, and human studies, while also highlighting current translational gaps in epigenetic validation across different metabolite classes.

## 6. Challenges, Limitations, and Future Perspectives

The transition from descriptive microbiota profiling to a mechanistic understanding of the MDME axis represents a significant step forward in nutritional sciences [10]. However, a critical synthesis of current literature reveals that while the epigenetic effects of certain metabolites, such as SCFAs, are well-documented, the evidence for other bioactives like peptides and polyphenols remains fragmented [12]. The broad bioactivity attributed to these compounds often overlooks the high inter-individual variability in microbial metabolic capacity, particularly regarding distinct metabotypes such as urolithin production and equol conversion [25,100], which complicates the reproducibility of epigenetic outcomes across different cohorts.

More critically, the field continues to operate under an implicit assumption that metabolite exposure produces uniform epigenetic responses, despite growing evidence that host-specific microbial architecture fundamentally determines metabolite bioavailability, transformation, and downstream signaling potential. This conceptual oversimplification remains one of the principal barriers to establishing predictive models of diet–microbiota–epigenome interactions.

### 6.1. Current Limitations of the Available Evidence

Despite the promising insights into the microbiota–epigenome axis, several limitations must be acknowledged to reduce potential bias. First, there is significant heterogeneity among microbiome studies, often stemming from differences in sequencing techniques, bioinformatic pipelines, and dietary intervention protocols [5].

This heterogeneity is further amplified by inconsistencies in intervention duration, substrate composition, dosage standardization, and participant dietary compliance, thereby limiting meaningful cross-study comparability and reducing confidence in generalized mechanistic inferences [19].

Such variability makes it challenging to establish a “universal” epigenetic signature for specific functional foods, as the same nutrient may yield different metabolites depending on the host’s baseline microbiota [101].

Furthermore, although animal models provide indispensable mechanistic insights, interspecies differences in microbial ecology, bile acid metabolism, and host epigenetic regulation frequently constrain direct translational relevance to human physiology [7,43]. Methodologically, current approaches are further limited by the dynamic and compartment-specific nature of metabolite pools; fecal and plasma measurements as surrogate markers often fail to accurately capture localized tissue-specific epigenetic activity [3].

Additional methodological constraints include limited cross-platform comparability in metabolomic quantification, insufficient temporal resolution to capture transient metabolite fluctuations, and the absence of standardized normalization frameworks for epigenomic analyses across tissues and analytical platforms [3,11].

Collectively, these methodological gaps reveal that the field has advanced more rapidly in descriptive analytical capacity than in experimental rigor, creating a disproportionate reliance on associative datasets that often exceed their mechanistic interpretive strength.

A deeper concern lies in the field’s continued reliance on correlative multi-omics associations without sufficient functional validation. While high-dimensional datasets have greatly expanded descriptive resolution, they have not proportionally advanced mechanistic certainty. As a result, much of the current literature risks conflating statistical association with biological causation.

Consequently, establishing causality remains a central unresolved challenge, as many studies fail to distinguish whether microbial alterations drive host epigenetic remodeling or merely emerge as secondary consequences of pre-existing host metabolic states [10,97].

This limitation reflects not merely a technical shortcoming, but a broader epistemological gap: the absence of experimental frameworks capable of disentangling bidirectional feedback loops within the MDME axis.

### 6.2. Future Research Directions

Addressing these challenges will require large-scale longitudinal human studies employing standardized analytical pipelines and harmonized intervention protocols to improve reproducibility across cohorts [19]. Integrative multi-omics approaches combining metagenomics, metabolomics, transcriptomics, and epigenomics at single-cell resolution will be essential for resolving the temporal and spatial complexity of nutrient–microbiota interactions and deciphering host responder versus non-responder phenotypes [3,11].

Future investigations should also prioritize strain-level functional resolution to clarify why individuals exhibit markedly divergent responses to identical dietary exposures [5,61].

In parallel, expanding research into host epigenetic memory may reveal how early-life microbial colonization programs long-term metabolic trajectories and disease susceptibility [9,52].

In addition, future research should broaden its scope to underexplored but biologically critical dimensions of host–microbiota–epigenome interactions. Sex-specific differences in microbial composition and epigenetic responsiveness remain insufficiently characterized, despite evidence suggesting differential metabolic and immune outcomes between males and females [7,91]. Similarly, early-life microbial and epigenetic programming, strongly influenced by maternal diet and perinatal exposures, represents a key determinant of long-term metabolic trajectories [52]. Moreover, drug–microbiota interactions may significantly modify metabolite bioavailability and epigenetic signaling pathways [36]. Dietary patterns characterized by ultra-processed food consumption further complicate this landscape by altering microbial diversity and reducing metabolite-driven regulatory capacity.

From a translational perspective, the implementation of precision nutrition is constrained by several practical limitations, including the lack of validated and reproducible clinical biomarkers for dynamic host–microbiome interactions [62], absence of standardized diagnostic endpoints across studies [62,80], high cost and infrastructural demands of multi-omics and longitudinal studies [6], and underdeveloped regulatory frameworks for microbiota-based interventions [4]. Ultimately, harnessing the MDME axis may enable the rational design of precision nutrition strategies tailored to individual host–microbiota configurations, marking a transition from descriptive nutrition science to predictive and systems-level therapeutic frameworks.

## 7. Conclusions

The diets comprise varied components, which act as precursors for epigenetic modulation mediated by the gut microbiota. In the microbiota–epigenome axis, fibers act as prebiotics for the sustainability and functionality of probiotics in gut microbiota. Fiber and polyphenols are metabolically transformed into bioactive compounds—such as SCFAs, urolithins, and phenolic acids—that act as a programmable interface directing host gene expression. Mechanistic insights, including SIRT1 activation, DNMT modulation via one-carbon metabolism, and targeted HDAC inhibition (e.g., CD36 upregulation), reveal how diet-microbiota interactions shape metabolic, inflammatory, and epigenetic outcomes.

Despite these advancements, key challenges remain to be further resolved. Inter-individual variability and metabotypes necessitate deciphering why only certain individuals efficiently convert precursors like ellagic acid into bioactive metabolites, a prerequisite for effective precision nutrition. Combinatory and synergistic effects of coexisting metabolites require further exploration, as interactions such as the “Butyrate–Polyphenol Synergy” may coordinate complex tissue-specific chromatin states. Stability and systemic impact of epigenetic modifications, including long-term memory and bioavailability in peripheral tissues, demand longitudinal studies and advanced pharmacokinetic modeling. Finally, the integration of multi-omics data—combining metagenomics, metabolomics, and epigenomics—will enable predictive frameworks for tailoring dietary interventions based on unique microbial signatures. By harnessing the gut microbiota as a programmable interface, agri-food by-products can transition from waste to precision tools for shaping human health. This integrative perspective provides a roadmap for microbiota-targeted, personalized nutritional strategies to prevent and manage metabolic and inflammatory diseases.

## Figures and Tables

**Table 1:** Functional Overview of Diet–Microbiota Interactions.

Dietary Component	Key Microbiota Effects	Representative Microbial Taxa/Key Metabolites	Primary Host Targets/Health Relevance	Predominantly Evidence-Based	Critical Notes/Limitations
Polyphenols	Microbial biotransformation into low-molecular-weight phenolics; modulation of microbial composition	*Eggerthella*, *Gordonibacter*/phenolic acids, urolithins	HDAC modulation, epithelial barrier reinforcement, anti-inflammatory signaling	Mixed translational evidence (strong mechanistic/preclinical; limited large-scale clinical validation)	Strong dependence on microbial enzymatic capacity; low intrinsic bioavailability without microbial conversion [14,22,30]
Flavonoids and Isoflavones	Enzymatic conversion into bioactive metabolites (e.g., equol)	*Slackia*, *Adlercreutzia*/(S)-equol	Estrogen receptor modulation, antioxidant defense, cardiometabolic regulation	Observational human evidence with small-scale intervention support	Only specific microbiota profiles enable metabolite production, resulting in high interindividual variability [25,35]
Dietary Lipids (PUFAs)	Indirect modulation via bile acids and host signaling pathways	*Bacteroides*, *Bilophila*/secondary bile acids	FXR/TGR5 signaling, inflammatory regulation, lipid homeostasis	Predominantly preclinical with emerging human validation	Effects depend on ω6/ω3 ratio; oxidative products may induce dysbiosis [28,33,41]
Saturated vs. Unsaturated Fats	High-fat diets reshape microbial composition and increase dysbiosis-associated taxa	Dysbiosis-associated microbial consortia/altered bile acid pools	Intestinal permeability, metabolic dysfunction, inflammatory signaling	Animal-supported with observational human corroboration	Outcomes are strongly context-dependent and influenced by baseline microbiota composition [40,49,50]
Microbial Lipid Metabolites (CLA, SCFAs)	Microbial conversion of fatty acids into bioactive lipid mediators	*Lactobacillus*, *Bifidobacterium*/CLA, acetate, butyrate	Immune modulation, chromatin accessibility, metabolic regulation	Mechanistically robust in vitro/animal evidence; heterogeneous clinical translation	Production is strain-specific and inconsistently reproducible across individuals [38,51]
Polysaccharides (General)	Fermentation by gut microbiota; selective enrichment of functional taxa	*Bacteroides*, *Prevotella*/SCFAs	Immune regulation, epithelial integrity, metabolic adaptation	Mixed mechanistic and translational evidence	Structural heterogeneity drives variable microbial responses and distinct metabolic outputs [15,17]
Dietary Fiber	Stimulation of SCFA-producing and beneficial bacteria	*Faecalibacterium prausnitzii*, *Roseburia*/butyrate, acetate, propionate	HDAC inhibition, epithelial barrier function, immune homeostasis	Strong human dietary intervention evidence with responder variability	Functional outcomes depend more on fermentability than absolute fiber quantity [15,30]
Resistant Starch	Selective enrichment of butyrate-producing taxa	*F. prausnitzii*, *Ruminococcus bromii*/butyrate	Metabolic regulation, colonic epithelial support	Controlled human intervention-supported	Marked responder/non-responder variability limits generalizability [17]
HMOs	Promotion of early-life beneficial taxa and cross-feeding networks	*Bifidobacterium infantis*, *B. bifidum*/acetate, lactate	Gut maturation, immune development, microbial ecosystem assembly	Strong mechanistic and clinical infant cohort evidence	Effects are age-dependent and dynamically regulated [52,53]
Cross-feeding Networks	Cooperative metabolic interactions among microbial taxa	Multi-species microbial consortia/SCFAs, vitamins	Amplified metabolite production and ecological resilience	Predominantly systems biology and in vitro evidence	Network complexity limits predictive modeling of outcomes [44]
Microbiota–Host Interaction	Modulation of host metabolic and immune pathways	Diverse community-derived metabolites	Homeostatic regulation and disease susceptibility modulation	Broad multi-level evidence base	Strong host–microbiome dependency introduces variability [7,9]
Microbiome and Epigenetics	Microbial metabolites regulate epigenetic pathways	SCFAs, bile acids, phenolic metabolites	DNA methylation, histone modification, transcriptional regulation	Predominantly mechanistic and associative evidence	Mechanistic pathways remain incompletely defined [10,11]
Microbiota and Immunotherapy	Modulation of response to PD-1-based therapies	Immunomodulatory microbial metabolites	Enhanced therapeutic efficacy and immune responsiveness	Early translational and observational clinical evidence	Outcomes are highly dependent on microbiota composition [35,47]

**Table 2:** Comparative Effects of Fiber Types on Microbial Metabolites and Cellular Targets.

Fiber Type	Solubility/Fermentability	Dominant Microbial Taxa	Key Metabolites Produced	Primary Host Cellular Target/Pathway	Epigenetic/Immune Relevance	Evidence Level	Critical Limitations
Inulin	Soluble, highly fermentable	*Bifidobacterium*, *Faecalibacterium*	SCFAs, secondary bile acids	TGR5, FXR, GPR43 signaling	Chromatin remodeling; immune modulation; type 2 inflammatory regulation	Animal + Human intervention	Strong responder/non-responder variability; dose-dependent outcomes [86]
Psyllium	Semi-soluble	*Bacteroides*, bile-acid-transforming taxa	SCFAs, bile acid derivatives	Tph1 regulation; enterochromaffin signaling	Anti-colitic effects; serotonin-mediated epithelial regulation	Animal studies	Limited mechanistic validation in humans [85]
Cellulose	Insoluble, poorly fermentable	Limited enrichment of fermentative taxa	Minimal metabolite generation	Weak receptor engagement	Minimal direct epigenetic modulation	Animal studies	Low fermentability restricts systemic signaling effects [86]
Resistant Starch	Fermentable	*Faecalibacterium prausnitzii*, *Ruminococcus bromii*	Butyrate, acetate	HDAC inhibition; 5-HT signaling	Enhanced metabolic homeostasis; anti-inflammatory regulation	Human observational + intervention	Marked interindividual metabolic variability [16,74]

Abbreviations: 2°BAs—Secondary Bile Acids. TGR5—Takeda G protein-coupled receptor 5. FXR—Farnesoid X Receptor. cAMP—Cyclic Adenosine Monophosphate. Tph1—Tryptophan Hydroxylase 1. EC cell—Enterochromaffin Cell. 5-HT—5-Hydroxytryptamine (Serotonin).

**Table 3:** SCFAs: Molecular Mechanisms, Cytological Targets, and Epigenetic Evidence.

SCFA	Dominant Microbial Producers	Receptors/Primary Mechanisms	Epigenetic Effects	Immune/Physiological Relevance	Evidence Level	Critical Limitations
Acetate	*Bacteroides* spp., *Bifidobacterium* spp.	GPR41, GPR43 activation; weak HDAC inhibition; acetyl-CoA precursor	Mild histone acetylation; contributes to acetyl-CoA pools	Supports CD8+ T-cell mitochondrial fitness; crosses BBB to influence neuroimmune signaling	Animal + mechanistic human studies	Rapid systemic turnover complicates tissue-specific quantification [16,77,81]
Propionate	*Bacteroides* spp., *Veillonella* spp.	GPR41/GPR43 activation; moderate HDAC inhibition	Locus-specific histone acetylation (e.g., FOXP3 regulation)	Promotes Treg differentiation; attenuates systemic inflammation	Animal + limited clinical intervention	High interindividual variation in production efficiency [16,83,93]
Butyrate	*Faecalibacterium prausnitzii*, *Roseburia* spp., Clostridium clusters IV/XIVa	GPR109A activation; potent HDAC inhibition; β-oxidation substrate	Strong histone acetylation; chromatin remodeling	Maintains epithelial hypoxia; regulates barrier integrity; induces apoptosis in neoplastic cells (“butyrate paradox”)	Extensive preclinical + selected human studies	Clinical translation remains inconsistent due to compartment-specific bioavailability [10,74,80,94]

Abbreviations: SCFAs: Short-Chain Fatty Acids. GPCRs: G-protein-coupled receptors. HDACi: Histone Deacetylase inhibitors. Kcr: Histone Crotonylation. OXPHOS: Oxidative Phosphorylation. Acetyl-CoA: Acetyl Coenzyme A. IECs: Intestinal Epithelial Cells. Treg: Regulatory T cells. BBB: Blood–Brain Barrier. CRC: Colorectal Cancer. MDMs: Microbiota-Derived Metabolites. FOXP3: Forkhead box P3 (transcription factor).

**Table 4:** Polyphenol-Derived MDMs: Microbial Transformation and Molecular pathways.

Polyphenol Class	Representative Microbial Metabolites	Dominant Microbial Dependency	Target Epigenetic Enzymes/Pathways	Cellular/Immune Relevance	Evidence Level	Critical Limitations
Ellagitannins	Urolithins (A, B)	Urolithin-producing consortia (metabotype-dependent)	SIRT1 activation; mitophagy induction	Enhanced mitochondrial respiration; reduced NF-κB signaling	In vitro + animal + limited human intervention	Strong responder/non-responder variability; incomplete pathway characterization [67,88]
Isoflavones	Equol	Specific equol-producing taxa	ERβ binding; DNMT modulation	Hormonal regulation; antioxidant vascular protection	Human observational + small intervention studies	Only a subset of individuals are equol producers [25,65]
Flavanols/Curcumin	Phenolic acids; tetrahydrocurcumin	Broad microbial reductive metabolism	HAT inhibition; Nrf2–Keap1 activation	ROS detoxification; anti-proliferative signaling	Predominantly in vitro/preclinical	Limited direct human mechanistic validation [95,96]
Anthocyanins	Protocatechuic acid	Microbiota-dependent phenolic degradation	Histone acetylation at inflammatory loci	Reduced IL-6/TNF-α production; immune modulation	Preclinical + observational human evidence	High metabolic instability and low reproducibility across cohorts [21,23]

Abbreviations: MDMs: Microbiota-Derived Metabolites. SIRT1: Sirtuin 1 (NAD-dependent deacetylase). NF-κB: Nuclear Factor kappa-light-chain-enhancer of activated B cells. ERβ: Estrogen Receptor beta. DNMT: DNA Methyltransferase. HAT: Histone Acetyltransferase. Nrf2: Nuclear factor erythroid 2-related factor 2. Keap1: Kelch-like ECH-associated protein 1. ROS: Reactive Oxygen Species. IL-6/TNF-α: Interleukin-6 and Tumor Necrosis Factor alpha.

## Data Availability

No new data were created or analyzed in this study.

## Abbreviations

The following abbreviations are used in this manuscript; the list is arranged in ABC order:

2°BAs	Secondary Bile Acids
5-HT	5-Hydroxytryptamine (Serotonin)
BBB	Blood–Brain Barrier
cAMP	Cyclic Adenosine Monophosphate
CAZymes	Carbohydrate-Active Enzymes
CD36	Cluster of Differentiation 36
DNA	Deoxyribonucleic Acid
EC cell	Enterochromaffin Cell
FXR	Farnesoid X Receptor
GABA	γ-aminobutyric acid
GPCRs	G protein-coupled receptors
GPR109A	G protein-coupled receptor 109A
GPR41	G protein-coupled receptor 41
GPR43	G protein-coupled receptor 43
HDAC6	Histone Deacetylase 6
HDACs	Histone Deacetylases
HFD	High-Fat Diet
HMOs	Human Milk Oligosaccharides
IL-6	Interleukin-6
KDM5	Lysine Demethylase 5
MCBAs	Microbially Conjugated Bile Acids
MDME Axis	Microbiota-Derived Metabolites–Epigenetic Axis
MDMs	Microbiota-Derived Metabolites
PUFAs	Polyunsaturated Fatty Acids
RNA	Ribonucleic Acid
SAM	S-adenosylmethionine
SCFAs	Short-Chain Fatty Acids
SIRT1	Sirtuin 1
TGR5	Takeda G protein-coupled receptor 5
TNF-α	Tumor Necrosis Factor alpha
Tph1	Tryptophan Hydroxylase 1

## References

[1] Terpou A., Dahiya D., Nigam P.S. (2025). Evolving interplay between fermented food microbiota and gut microenvironment—Strategic pathways to improve human health. Foods.

[2] Rubas N.C., Torres A., Maunakea A.K. (2025). The gut microbiome and epigenomic reprogramming: Mechanisms, interactions, and implications for human health and disease. Int. J. Mol. Sci..

[3] Dahiya D., Nigam P.S. (2023). Therapeutic and Dietary Support for Gastrointestinal Tract Using Kefir as a Nutraceutical Beverage: Dairy-Milk-Based or Plant-Sourced Kefir Probiotic Products for Vegan and Lactose-Intolerant Populations. Fermentation.

[4] Edkaidek H., Dahiya D., Nigam P.S. (2026). Prospects of Bioactive Compounds in Designing Functional Foods: Challenges and Solutions. Foods.

[5] Dahiya D., Terpou A., Nigam P.S. (2025). Strategic Restoration of Sustainability in Gut Microbiome Diversity Through Synbiotic Food, Functional Beverages or Commercial Probiotic Formulations: Achieving Sustainable Development Goals 03, 09 and 12. SCI Sustain..

[6] Zhernakova D.V., Wang D., Liu L., Andreu-Sánchez S., Zhang Y., Ruiz-Moreno A.J., Peng H., Plomp N., Del Castillo-Izquierdo Á., Gacesa R. (2024). Host genetic regulation of human gut microbial structural variation. Nature.

[7] Lee J., Tsolis R., Bäumler A. (2022). The microbiome and gut homeostasis. Science.

[8] Hansen S.B., Bozzi D., Mak S.S., Clausen C.G., Nielsen T.K., Kodama M., Hansen L.H., Gilbert M.T.P., Limborg M.T. (2023). Intestinal epigenotype of Atlantic salmon (*Salmo salar*) associates with tenacibaculosis and gut microbiota composition. Genomics.

[9] Pepke M.L., Hansen S.B., Limborg M.T. (2024). Unraveling host regulation of gut microbiota through the epigenome–microbiome axis. Trends Microbiol..

[10] Lin X., Han H., Wang N., Wang C., Qi M., Wang J., Liu G. (2024). The gut microbial regulation of epigenetic modification from a metabolic perspective. Int. J. Mol. Sci..

[11] Lei J., Wang X., Liu X. (2025). Microbiota-derived metabolites in the epigenetic regulation of the host. Sci. Bull..

[12] Williamson G. (2025). Bioavailability of food polyphenols: Current state of knowledge. Annu. Rev. Food Sci. Technol..

[13] Chen L., Cao H., Huang Q., Xiao J., Teng H. (2022). Absorption, metabolism and bioavailability of flavonoids: A review. Crit. Rev. Food Sci. Nutr..

[14] Alqudah S., Claesen J. (2024). Mechanisms of gut bacterial metabolism of dietary polyphenols into bioactive compounds. Gut Microbes.

[15] Zheng Y., Qin C., Wen M., Zhang L., Wang W. (2024). The effects of food nutrients and bioactive compounds on the gut microbiota: A comprehensive review. Foods.

[16] Mann E.R., Lam Y.K., Uhlig H.H. (2024). Short-chain fatty acids: Linking diet, the microbiome and immunity. Nat. Rev. Immunol..

[17] Deehan E.C., Zhang Z., Riva A., Armet A.M., Perez-Muñoz M.E., Nguyen N.K., Krysa J.A., Seethaler B., Zhao Y.-Y., Cole J. (2022). Elucidating the role of the gut microbiota in the physiological effects of dietary fiber. Microbiome.

[18] Zhan Q., Wang R., Thakur K., Feng J.-Y., Zhu Y.-Y., Zhang J.-G., Wei Z.-J. (2024). Unveiling of dietary and gut-microbiota derived B vitamins: Metabolism patterns and their synergistic functions in gut-brain homeostasis. Crit. Rev. Food Sci. Nutr..

[19] Luo J., Wang Y. (2025). Precision dietary intervention: Gut microbiome and meta-metabolome as functional readouts. Phenomics.

[20] Chau T.C., Owusu-Apenten R., Nigam P.S. (2017). Total Phenols, Antioxidant Capacity and Antibacterial Activity of Manuka Honey Extract. J. Adv. Biol. Biotechnol..

[21] Kirkpatrick G., Nigam P., Owusu-Apenten R.K. (2017). Total Phenols, Antioxidant Capacity and Antibacterial Activity of Manuka Honey Chemical Constituents. J. Adv. Biol. Biotechnol..

[22] Li Z., Kanwal R., Yue X., Li M., Xie A. (2024). Polyphenols and intestinal microorganisms: A review of their interactions and effects on human health. Food Biosci..

[23] Li H., Gao J.a., Peng W., Sun X., Qi W., Wang Y. (2025). Dietary Polyphenols-Gut Microbiota Interactions: Intervention Strategies and Metabolic Regulation for Intestinal Diseases. Biology.

[24] Ruiz de la Bastida A., Langa S., Curiel J.A., Peirotén Á., Landete J.M. (2024). Effect of fermented soy beverage on equol production by fecal microbiota. Foods.

[25] Lv J., Jin S., Zhang Y., Zhou Y., Li M., Feng N. (2024). Equol: A metabolite of gut microbiota with potential antitumor effects. Gut Pathog..

[26] Grek O., Tymchuk A., Soloviov N., Shumylo O., Bogachuk A. (2025). Antioxidant effects of plant ingredients on a dairy lipid base. Ukr. Food J..

[27] Ballini A., Barile S.N., De Rosa A., Bizzoca M.E., Boccellino M., Scacco S., Cantore S., Lo Muzio L., Lasorsa F.M., Arrigoni R. (2026). The Polyphenol–Microbiota Axis: Molecular Mechanisms, Metabolic Pathways, and Therapeutic Perspectives in Human Health. J. Pers. Med..

[28] Du S., Li R., Liu Y., Yang W., Ye Z. (2025). High ω6/ω3 polyunsaturated fatty acid (PUFA) ratio impairs intestinal mucosal barrier function via ROS/TLR4/NF-κB-mediated aberrant expression of tight junction proteins. Food Biosci..

[29] Hays K.E., Pfaffinger J.M., Ryznar R. (2024). The interplay between gut microbiota, short-chain fatty acids, and implications for host health and disease. Gut Microbes.

[30] Mohammadi F., Rudkowska I. (2025). Dietary lipids, gut microbiota, and their metabolites: Insights from recent studies. Nutrients.

[31] Mushtaq M., Sultana B., Akram S., Adnan A., Owusu-Apenten R.K., Nigam P. (2016). Enzyme-assisted Extraction of Polyphenols from Pomegranate (*Punica granatum*) Peel. J. Microbiol. Biotechnol..

[32] Schoeler M., Caesar R. (2019). Dietary lipids, gut microbiota and lipid metabolism. Rev. Endocr. Metab. Disord..

[33] Watanabe M., Fujita Y., Hagio M., Ishizuka S., Ogura Y., Hayashi T., Fukiya S., Yokota A. (2025). Bile acid is a responsible host factor for high-fat diet-induced gut microbiota alterations in rats: Proof of the “bile acid hypothesis”. Biosci. Microbiota Food Health.

[34] Umemura M., Honda A., Yamashita M., Chida T., Noritake H., Yamamoto K., Honda T., Ichimura-Shimizu M., Tsuneyama K., Miyazaki T. (2024). High-fat diet modulates bile acid composition and gut microbiota, affecting severe cholangitis and cirrhotic change in murine primary biliary cholangitis. J. Autoimmun..

[35] Bautista J., Lamas-Maceiras M., Hidalgo-Tinoco C., Guerra-Guerrero A., Betancourt-Velarde A., López-Cortés A. (2026). Gut microbiome–driven colorectal cancer via immune, metabolic, neural, and endocrine axes reprogramming. npj Biofilms Microbiomes.

[36] Agus A., Clément K., Sokol H. (2021). Gut microbiota-derived metabolites as central regulators in metabolic disorders. Gut.

[37] Jayapala H.P., Lim S.Y. (2023). N-3 polyunsaturated fatty acids and gut microbiota. Comb. Chem. High Throughput Screen..

[38] Nasrollahzadeh A., Tavani S.M., Arjeh E., Jafari S.M. (2023). Production of conjugated linoleic acid by lactic acid bacteria; important factors and optimum conditions. Food Chem. X.

[39] Chen P.-Y., Yeh Y.-M., Chen C.-H., Li M.-C., Hsu Y.-T., Huang P.-J., Cheng W.-H. (2026). *Bifidobacterium breve* promotes growth and lipid alteration in *Trichomonas vaginalis* transiently through transcriptomic reprogramming. Sci. Rep..

[40] Zhu T., Kuai Y., Guo X., Bu G., Yang C., Chen F. (2024). Effect of Dietary Oils with Different Fatty Acid Compositions on Serum Lipid and Gut Microbiota of Rats. Foods.

[41] Ruan M., Zhang Z., Yuan X., Zhou R., Zhang S., Tian Y., Li X., Li N., Liu Z., Zhu R. (2023). Effects of deep frying vegetable oils rich in PUFAs on gut microbiota in rats. Int. J. Food Sci. Technol..

[42] Wei Y., Luo J., Zhang L., Zhang L., Huang X., Chai Y., Wang W., Yan Q., Qiu Y., Li B. (2025). The potential of flavonoids as microbiota-directed foods regulating lipid metabolism via the gut-liver axis. Food Sci. Hum. Wellness.

[43] Lee M.H., Nuccio S.-P., Mohanty I., Hagey L.R., Dorrestein P.C., Chu H., Raffatellu M. (2024). How bile acids and the microbiota interact to shape host immunity. Nat. Rev. Immunol..

[44] Culp E.J., Goodman A.L. (2023). Cross-feeding in the gut microbiome: Ecology and mechanisms. Cell Host Microbe.

[45] Dahiya D., Nigam P.S. (2026). Application of Suitable Bioactive Probiotic Strains Sustaining Gut Microflora for Healthcare and Disease Prevention. Appl. Sci..

[46] Catalkaya G., Venema K., Lucini L., Rocchetti G., Delmas D., Daglia M., De Filippis A., Xiao H., Quiles J.L., Xiao J. (2020). Interaction of dietary polyphenols and gut microbiota: Microbial metabolism of polyphenols, influence on the gut microbiota, and implications on host health. Food Front..

[47] Huang J., Liu D., Wang Y., Liu L., Li J., Yuan J., Jiang Z., Jiang Z., Hsiao W.W., Liu H. (2022). Ginseng polysaccharides alter the gut microbiota and kynurenine/tryptophan ratio, potentiating the antitumour effect of antiprogrammed cell death 1/programmed cell death ligand 1 (anti-PD-1/PD-L1) immunotherapy. Gut.

[48] Betrouche A., Estivi L., Colombo D., Pasini G., Benatallah L., Brandolini A., Hidalgo A. (2022). Antioxidant properties of gluten-free pasta enriched with vegetable by-products. Molecules.

[49] Salsinha A.S., Araújo-Rodrigues H., Dias C., Cima A., Rodríguez-Alcalá L.M., Relvas J.B., Pintado M. (2025). Omega-3 and conjugated fatty acids impact on human microbiota modulation using an in vitro fecal fermentation model. Clin. Nutr..

[50] Zapata J., Gallardo A., Romero C., Valenzuela R., Garcia-Diaz D., Duarte L., Bustamante A., Gasaly N., Gotteland M., Echeverria F. (2022). n-3 polyunsaturated fatty acids in the regulation of adipose tissue browning and thermogenesis in obesity: Potential relationship with gut microbiota. Prostaglandins Leukot. Essent. Fat. Acids.

[51] Ney L.-M., Wipplinger M., Grossmann M., Engert N., Wegner V.D., Mosig A.S. (2023). Short chain fatty acids: Key regulators of the local and systemic immune response in inflammatory diseases and infections. Open Biol..

[52] Lordan C., Roche A.K., Delsing D., Nauta A., Groeneveld A., MacSharry J., Cotter P.D., van Sinderen D. (2024). Linking human milk oligosaccharide metabolism and early life gut microbiota: Bifidobacteria and beyond. Microbiol. Mol. Biol. Rev..

[53] Onodera H., Sato Y., Komatsu Y., Yamashita M., Watanabe Y., Kokubo T. (2025). HMOs induce butyrate production of Faecalibacterium prausnitzii via cross-feeding by Bifidobacterium bifidum with different mechanisms for HMO types. Microorganisms.

[54] Fu J., Zheng Y., Gao Y., Xu W. (2022). Dietary fiber intake and gut microbiota in human health. Microorganisms.

[55] Grant E.T., De Franco H., Desai M.S. (2025). Non-SCFA microbial metabolites associated with fiber fermentation and host health. Trends Endocrinol. Metab..

[56] Zhang B., Zhang Y., Xing X., Wang S. (2022). Health benefits of dietary polyphenols: Insight into interindividual variability in absorption and metabolism. Curr. Opin. Food Sci..

[57] Dahiya D., Nigam P. (2023). Use of Characterized Microorganisms in Fermentation of Non-Dairy-Based Substrates to Produce Probiotic Food for Gut-Health and Nutrition. Fermentation.

[58] Dahiya D., Nigam P.S. (2023). Nutraceutical Combinational Therapy for Diarrhoea Control with Probiotic Beverages from Fermented Fruits, Vegetables and Cereals to Regain Lost Hydration, Nutrition and Gut Microbiota. Microorganisms.

[59] Mousavi S., Ashooriyan P., Rostami M.Y., Erfani M., Nigam P.S. (2026). Green Sustainable Synthesis of Silver Nanoparticles from Extracts of Garden Spearmint Mentha spicata for Antibiotic and Antioxidant Activities. Sustain. Process. Connect.

[60] Zhang X., Wang Y., Li Z., Li Y., Qi B. (2024). Effects of polysaccharide type on the structure, interface behavior, and foam properties of soybean protein isolate hydrolysate-polysaccharide Maillard conjugates. Food Hydrocoll..

[61] Wardman J.F., Bains R.K., Rahfeld P., Withers S.G. (2022). Carbohydrate-active enzymes (CAZymes) in the gut microbiome. Nat. Rev. Microbiol..

[62] Simon M.C., Sina C., Ferrario P.G., Daniel H., Society W.G.P.N.o.t.G.N. (2023). Gut microbiome analysis for personalized nutrition: The state of science. Mol. Nutr. Food Res..

[63] Salvo A., Masciulli F., Ambroselli D., Romano E., Ingallina C., Spano M., Di Matteo G., Giusti A.M., Di Sotto A., Percaccio E. (2025). Hydrolysates from cauliflower and artichoke industrial wastes as biostimulants on seed germination and seedling growth: A chemical and biological characterization. J. Sci. Food Agric..

[64] Mohammadnezhad P., Valdés A., Álvarez-Rivera G. (2023). Bioactivity of food by-products: An updated insight. Curr. Opin. Food Sci..

[65] Gong Y., Lv J., Pang X., Zhang S., Zhang G., Liu L., Wang Y., Li C. (2023). Advances in the metabolic mechanism and functional characteristics of equol. Foods.

[66] Garcia-Villalba R., Vissenaekens H., Pitart J., Romo-Vaquero M., Espin J.C., Grootaert C., Selma M.V., Raes K., Smagghe G., Possemiers S. (2017). Gastrointestinal simulation model TWIN-SHIME shows differences between human urolithin-metabotypes in gut microbiota composition, pomegranate polyphenol metabolism, and transport along the intestinal tract. J. Agric. Food Chem..

[67] Garcia-Villalba R., Giménez-Bastida J.A., Cortés-Martín A., Ávila-Gálvez M.Á., Tomás-Barberán F.A., Selma M.V., Espín J.C., González-Sarrías A. (2022). Urolithins: A comprehensive update on their metabolism, bioactivity, and associated gut microbiota. Mol. Nutr. Food Res..

[68] Machado M., Silva S., Costa E.M. (2024). Byproducts as a sustainable source of cosmetic ingredients. Appl. Sci..

[69] Das P., Babaei P., Nielsen J. (2019). Metagenomic analysis of microbe-mediated vitamin metabolism in the human gut microbiome. BMC Genom..

[70] Gaur G., Oh J.-H., Filannino P., Gobbetti M., Van Pijkeren J.-P., Gänzle M.G. (2020). Genetic determinants of hydroxycinnamic acid metabolism in heterofermentative lactobacilli. Appl. Environ. Microbiol..

[71] Reguengo L.M., Salgaço M.K., Sivieri K., Júnior M.R.M. (2022). Agro-industrial by-products: Valuable sources of bioactive compounds. Food Res. Int..

[72] Wang J., Zhang Y., Wu Q., Zhong Y., Xu Z., Yang J. (2026). Interactions of bile acids and gut microbiota modulate neurological health: A comprehensive review on mechanisms and therapeutic potential of dietary phytochemicals. Front. Microbiol..

[73] Zeng L., Qian Y., Cui X., Zhao J., Ning Z., Cha J., Wang K., Ge C., Jia J., Dou T. (2025). Immunomodulatory role of gut microbial metabolites: Mechanistic insights and therapeutic frontiers. Front. Microbiol..

[74] Mukhopadhya I., Louis P. (2025). Gut microbiota-derived short-chain fatty acids and their role in human health and disease. Nat. Rev. Microbiol..

[75] Kim C.-S. (2024). Roles of diet-associated gut microbial metabolites on brain health: Cell-to-cell interactions between gut bacteria and the central nervous system. Adv. Nutr..

[76] Berding K., Carbia C., Cryan J.F. (2021). Going with the grain: Fiber, cognition, and the microbiota-gut-brain-axis. Exp. Biol. Med..

[77] Zhang D., Jian Y.-P., Zhang Y.-N., Li Y., Gu L.-T., Sun H.-H., Liu M.-D., Zhou H.-L., Wang Y.-S., Xu Z.-X. (2023). Short-chain fatty acids in diseases. Cell Commun. Signal..

[78] Stanimirov B., Đanić M., Pavlović N., Zaklan D., Lazarević S., Mikov M., Stankov K. (2025). Gut–Brain Axis and Bile Acid Signaling: Linking Microbial Metabolism to Brain Function and Metabolic Regulation. Int. J. Mol. Sci..

[79] Rudrapal M., de Oliveira A.M., Singh R.P. (2025). Dietary polyphenols maintain human health through modulation of gut microbiota. Front. Pharmacol..

[80] Wang J., Zhao Q., Zhang S., Liu J., Fan X., Han B., Hou Y., Ai X. (2025). Microbial short chain fatty acids: Effective histone deacetylase inhibitors in immune regulation. Int. J. Mol. Med..

[81] Dahiya D., Nigam P. (2022). Nutrition and Health through the Use of Probiotic Strains in Fermentation to Produce Non-Dairy Functional Beverage Products Supporting Gut Microbiota. Foods.

[82] Nireeksha, Maniangat Luke A., Kumari N S., Hegde M.N., Hegde N.N. (2025). Metabolic interplay of SCFA’s in the gut and oral microbiome: A link to health and disease. Front. Oral Health.

[83] Prado C., Espinoza A., Martínez-Hernández J.E., Petrosino J., Riquelme E., Martin A.J., Pacheco R. (2023). GPR43 stimulation on TCRαβ+ intraepithelial colonic lymphocytes inhibits the recruitment of encephalitogenic T-cells into the central nervous system and attenuates the development of autoimmunity. J. Neuroinflamm..

[84] Shimizu H., Masujima Y., Ushiroda C., Mizushima R., Taira S., Ohue-Kitano R., Kimura I. (2019). Dietary short-chain fatty acid intake improves the hepatic metabolic condition via FFAR3. Sci. Rep..

[85] Bretin A., Zou J., San Yeoh B., Ngo V.L., Winer S., Winer D.A., Reddivari L., Pellizzon M., Walters W.A., Patterson A.D. (2023). Psyllium fiber protects against colitis via activation of bile acid sensor farnesoid X receptor. Cell. Mol. Gastroenterol. Hepatol..

[86] Arifuzzaman M., Won T.H., Li T.-T., Yano H., Digumarthi S., Heras A.F., Zhang W., Parkhurst C.N., Kashyap S., Jin W.-B. (2022). Inulin fibre promotes microbiota-derived bile acids and type 2 inflammation. Nature.

[87] Dahiya D., Nigam P.S. (2023). Inclusion of Dietary-Fibers in Nutrition Provides Prebiotic Substrates to Probiotics for the Synthesis of Beneficial Metabolites SCFA to Sustain Gut Health Minimizing Risk of IBS, IBD, CRC. Recent Prog. Nutr..

[88] Hasheminezhad S.H., Boozari M., Iranshahi M., Yazarlu O., Sahebkar A., Hasanpour M., Iranshahy M. (2022). A mechanistic insight into the biological activities of urolithins as gut microbial metabolites of ellagitannins. Phytother. Res..

[89] Choudhuri S., Klaassen C.D. (2022). Molecular regulation of bile acid homeostasis. Drug Metab. Dispos..

[90] Guzior D.V., Quinn R.A. (2021). Microbial transformations of human bile acids. Microbiome.

[91] Fan Y., Pedersen O. (2021). Gut microbiota in human metabolic health and disease. Nat. Rev. Microbiol..

[92] Dahiya D., Nigam P. (2022). Probiotics, Prebiotics, Synbiotics, and Fermented Foods as potential biotics in Nutrition Improving Health via Microbiome-Gut-Brain Axis. Fermentation.

[93] Pang A., Pu S., Pan Y., Huang N., Li D. (2025). Short-chain fatty acids from gut microbiota restore Th17/Treg balance in rheumatoid arthritis: Mechanisms and therapeutic potential. J. Transl. Autoimmun..

[94] Dahiya D., Nigam P.S. (2023). Biotherapy Using Probiotics as Therapeutic Agents to Restore the Gut Microbiota to Relieve Gastrointestinal Tract Inflammation, IBD, IBS and Prevent Induction of Cancer. Int. J. Mol. Sci..

[95] Aldhirgham T., Henderson K., Nigam P., Owusu-Apenten R.K. (2016). A Combination of Curcumin from Turmeric and Alpha-linolenic Acid Shows Antagonism with MCF-7 Breast Cancer Cells in Phenol-red Free Medium. J. Appl. Life Sci. Int..

[96] Cheung K.N., Nigam P.S., Owusu-Apenten R. (2016). Antioxidant Activity of Curcumin and Neem (*Azadirachta indica*) Powders: Combination Studies with ALA Using MCF-7 Breast Cancer Cells. J. Appl. Life Sci. Int..

[97] Dahiya D., Nigam P. (2022). The Gut Microbiota Influenced by the Intake of Probiotics and Functional Foods with Prebiotics Can Sustain Wellness and Alleviate Certain Ailments like Gut-Inflammation and Colon-Cancer. Microorganisms.

[98] Ku K., Park I., Kim D., Kim J., Jang S., Choi M., Choe H.K., Kim K. (2020). Gut microbial metabolites induce changes in circadian oscillation of clock gene expression in the mouse embryonic fibroblasts. Mol. Cells.

[99] Kim J., Lee H.K. (2021). The role of gut microbiota in modulating tumor growth and anticancer agent efficacy. Mol. Cells.

[100] Hu J., Mesnage R., Tuohy K., Heiss C., Rodriguez-Mateos A. (2024). (Poly)phenol-related gut metabotypes and human health: An update. Food Funct..

[101] Edkaidek H., Dahiya D., Nigam P.S. (2026). Characteristics and health benefits of the probiotic yeast *Saccharomyces boulardii*. Microbes Immun..

